# Management of an Intraparenchymal Struvite Stone Using Laparoscopic Nephrolithotomy: A Case Report

**DOI:** 10.1002/ccr3.70065

**Published:** 2025-01-06

**Authors:** Ayoub Hidayat‐Allah, Frank Friedersdorff

**Affiliations:** ^1^ Department of Urology Evangelisches Krankenhaus Königin Elisabeth Herzberge gGmbH Berlin Germany; ^2^ Department of Urology Charite Universitatsmedizin Berlin Berlin Germany

**Keywords:** case report, laparoscopic nephrolithotomy, nephrolithiasis, struvite stones

## Abstract

This study aims to present a case of laparoscopic nephrolithotomy and highlight its successful outcome. The patient was a 65‐year‐old male who experienced intermittent colicky flank pain. Imaging revealed the presence of a 20‐mm round‐shaped stone in a hydronephrotic calyx located in the mid‐pole of the left kidney, possibly with a parenchymal component. Given the stone's location, neither percutaneous nephrolithotripsy (PCNL) nor ureterorenoscopy (URS) was considered feasible. The findings of this study demonstrate the effectiveness and suitability of laparoscopic nephrolithotomy (LNL) in managing stones. It emphasizes that LNL can be utilized not only as an alternative treatment but also as a viable initial option when appropriate case selection is made.

**Summary**

- The laparoscopic approach should be considered for stone treatment when mini‐PNL and URS are not viable options.
- Conduct a metabolic analysis of the stones in all cases.
- Infection stones are infrequent, yet they pose greater risks and challenges during treatment.

## 1 Introduction

For managing large upper impacted ureteral stones, three minimally invasive methods are to date available: percutaneous nephrolithotomy (PCNL), transurethral ureteroscope lithotripsy (URSL) and laparoscopic pyelolithotomy (LPL). Laparoscopic stone removal is typically reserved for special cases and has been reported in only a limited number of studies. LPL can sometimes be considered as an alternative to URS, shock wave lithotripsy (SWL), or PNL for large proximal ureteral stones.

In accordance with the recommendations of the European Association of Urology (EAU), laparoscopic or open surgical stone removal should be offered in rare cases where shock wave lithotripsy, retrograde or antegrade ureteroscopy, and percutaneous nephrolithotomy are unsuccessful or unlikely to succeed [1].

This case report describes a 65‐year‐old man who initially presented with intermittent colicky flank pain. Computed tomography (CT) scanning revealed a round‐shaped stone measuring 17 × 17 × 20 mm in a hydronephrotic calyx located in the mid‐pole of the left kidney, potentially with a parenchymal component. The patient underwent successful treatment with laparoscopic nephrolithotomy.

## 2 Case History

A 65‐year‐old male patient in good overall health was referred to our department by his local urologist with a significant left renal parenchymal calcification in the setting of known recurrent nephrolithiasis, with a history of multiple sessions of PNL and URS performed bilaterally. The patient reports intermittent flank pain for several months.

His previous medical history included, in addition to recurrent bilateral urolithiasis, arterial hypertonia, and an appendectomy, but no significant medical issues. The physical examination was unremarkable. His body mass index was 35.19 kg/m^2^. The Patient scored 0 in the Eastern Cooperative Oncology Group (ECOG) Performance Score, and I in the American Society of Anaesthesiologists (ASA) physical status classification. Renal function tests performed before the intervention showed a mildly decreased glomerular filtration rate (GFR) (66 mL/min/1.73 m^2^).

## 3 Methods

**FIGURE 1:**
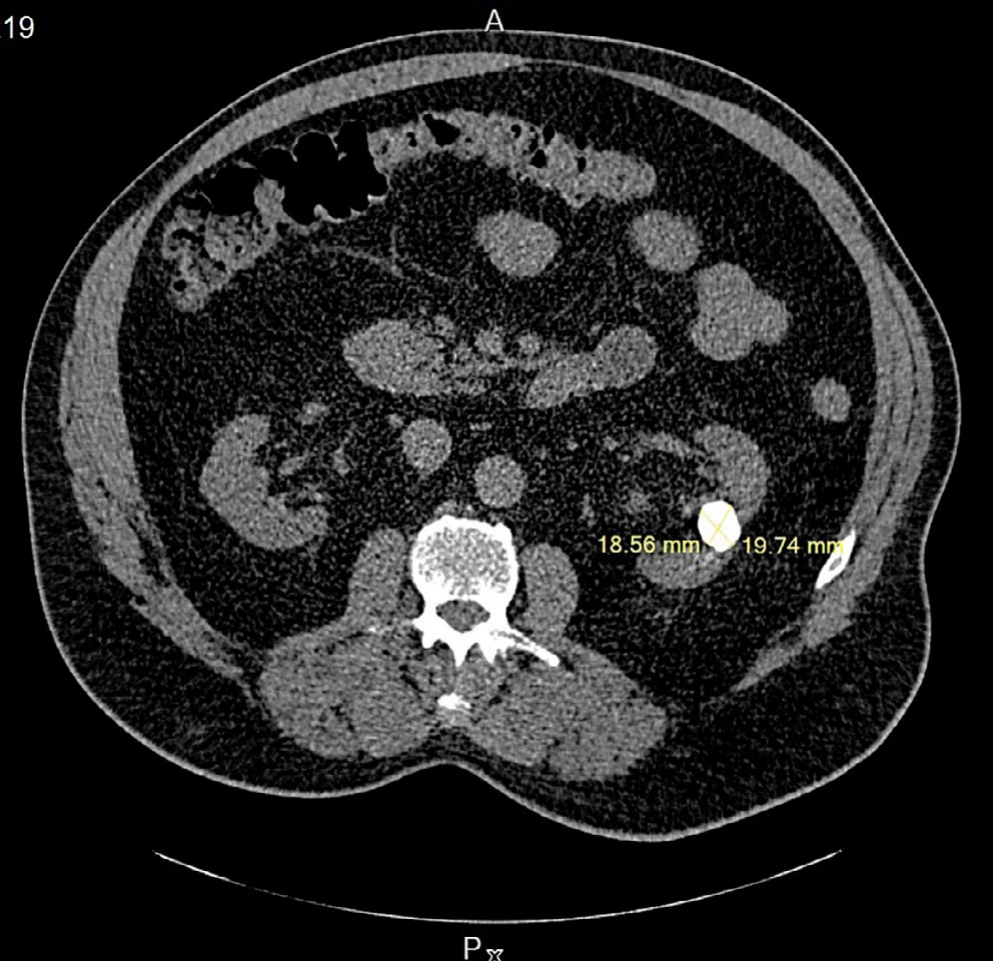
Axial plan of an abdominal computer tomography showing the round‐shaped stone in a hydronephrotic calyx in the mid‐pole of the left kidney before the laparoscopic nephrolithotomy.

**FIGURE 2:**
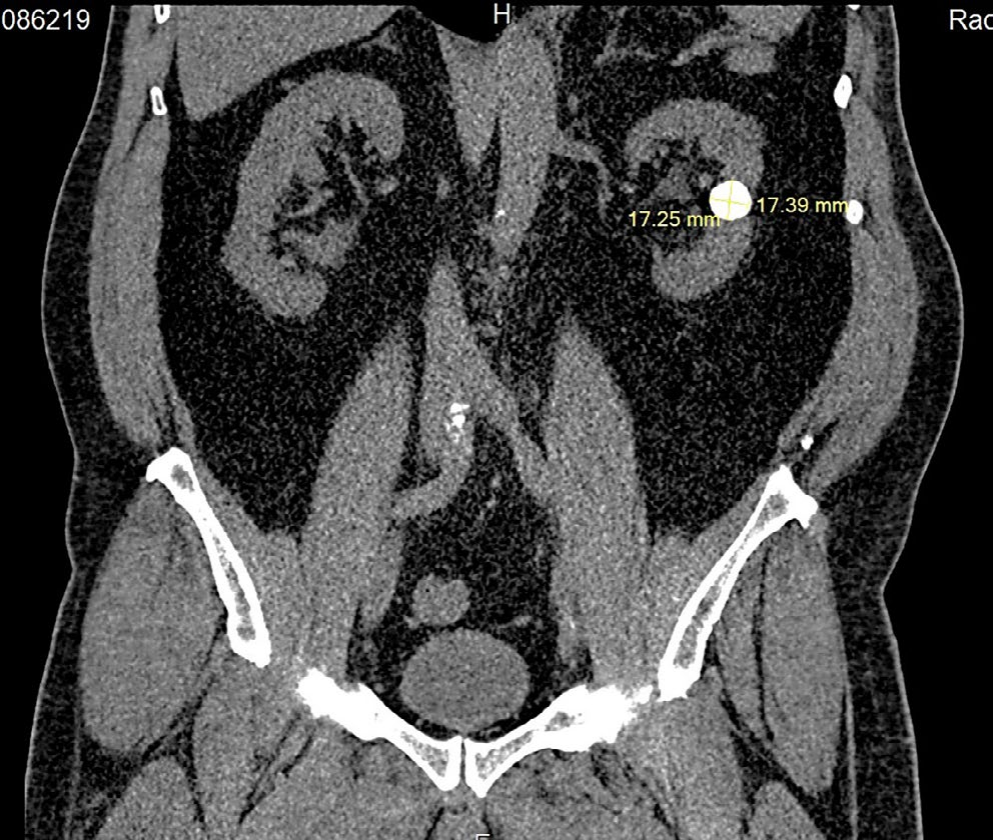
Coronal plan of an abdominal computer tomography showing the round‐shaped stone in a hydronephrotic calyx in the mid‐pole of the left kidney before the laparoscopic nephrolithotomy.

To further investigate the nephrolithiasis detected by ultrasound, a CT scan was performed. It showed orthotopic, normally sized kidneys with no hydronephrosis, 17 × 17 × 20 mm round‐shaped stone in a hydronephrotic calyx in the mid‐pole of the left kidney, possibly parenchymal calcification, 13 mm stone in the lower calyx group on the left, as well as two stones measuring up to 6 mm in the lower calyx group on the right. There was no evidence of stones along the ureters and in the urinary bladder (Figures 1 and 2).

Following a thorough discussion with the patient and a comprehensive evaluation of the various risks and benefits involved, we decided to proceed with an elective laparoscopic nephrolithotomy. Due to the location of the calcifications, a percutaneous nephrolithotripsy was not deemed feasible.

After administering a parenteral single‐shot antibiotic prophylaxis with 3 g of Sultamicillin and conducting a team timeout in the operating room, the patient was positioned in a slightly extended right lateral decubitus position with appropriate padding.

A 12‐mm incision was made below the umbilicus on the left side, and pneumoperitoneum was established at 15 mmHg. Once a 12‐mm trocar was inserted, a 3D optics system was utilized to gain an overview, revealing no injuries. Extensive adhesions were observed in the left upper and lower abdomen, necessitating adhesiolysis. A 12‐mm trocar was inserted in the left lower abdomen, accompanied by a 5‐mm trocar in the upper abdomen.

During the procedure, it was noted that the spleen was completely deviated to the left due to fibrous fat, and Gerota's fascia was firmly adhered to the lateral abdominal wall. The pancreas was found adjacent to the kidney, and the descending colon was mobilized as required. Attention was focused on the renal capsule.

**FIGURE 3:**
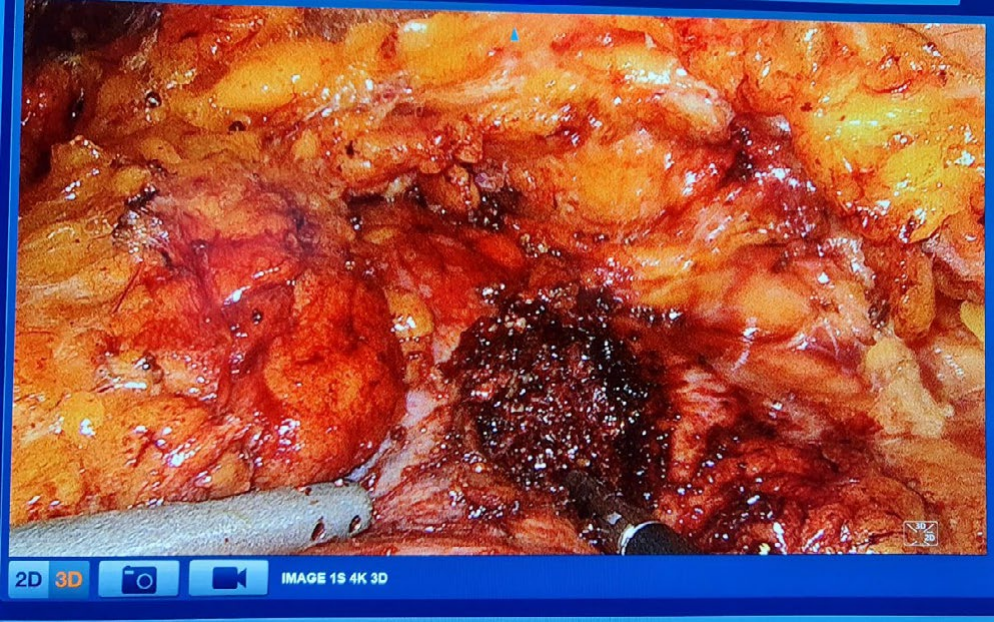
Intraoperative laparoscopic view of the stone.

**FIGURE 4:**
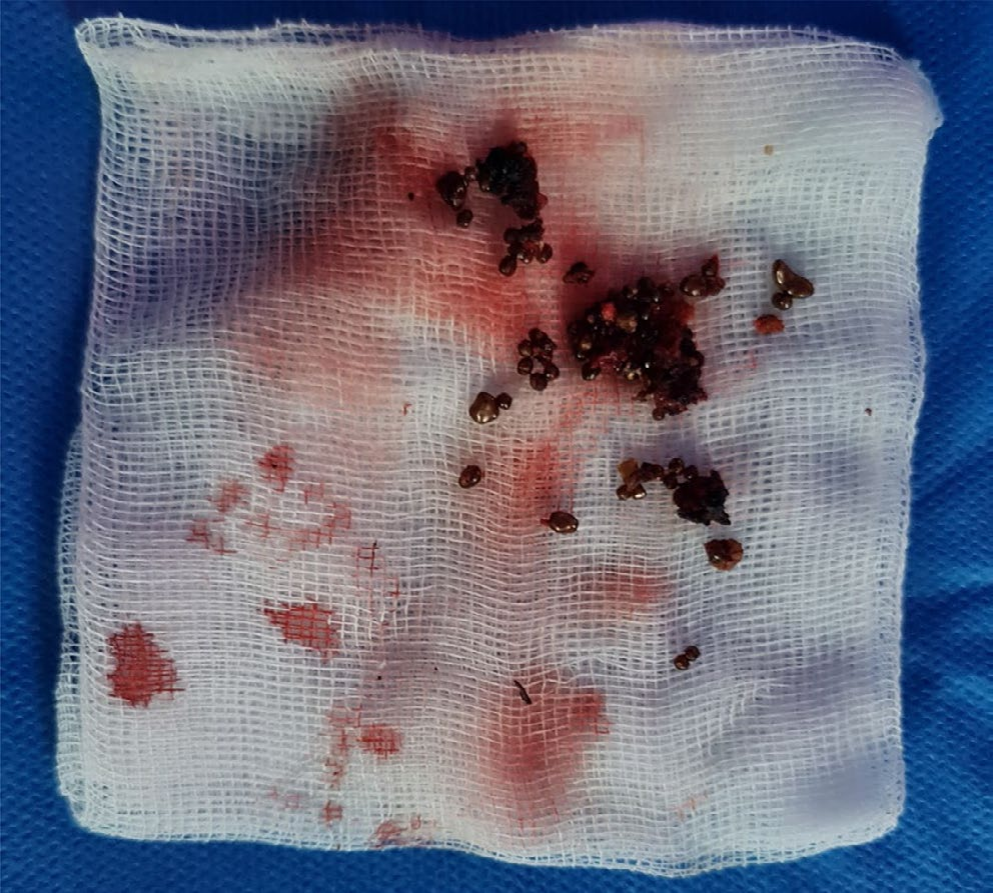
Removed fragments of the struvite calculi.

Following the removal of fibrous tissue, a hardened area in the middle region was identified and partially resected. The stone located at the deepest point within the kidney parenchyma was soft and fragmented (Figure 3). The fragments were successfully retrieved (Figure 4). Coagulation of the parenchymal defect was performed, and Hemopatch, Resorbacell roll, and 4 Dry Fields were employed to ensure hemostasis. No significant bleeding was observed. The estimated blood loss during the procedure amounted to approximately 50 mL.

A purse‐string suture was applied, and a 15 French Robinson drainage tube was inserted into the left renal bed. The trocars were removed under direct vision, and fascial closure, skin sutures, and sterile wound dressing were applied.

## 4 Conclusion and Results

The patient's hospitalization, including the period with the drainage in place, lasted for a total of 5 days. No complications related to the procedure were observed during this time. The drainage fluid was tested for creatinine and lipase levels, which were found to be consistent with the levels in the serum. Consequently, the drainage was removed. The suture material utilized was self‐absorbable, and the wounds remained non‐irritating throughout the recovery process.

The metabolic analysis of the stone revealed an infection stone with a composition of 50% Magnesium‐Ammoniumphosphat‐Hexahydrat (struvite), 30% Mono‐Ammoniumurat, and 20% Calcium‐Carbonat‐Phostat (Carbonatapatit). Considering the severity of the findings, it was recommended to undergo an antibiotic long‐term prophylaxis and a complete removal of the remaining stones. Additionally, urinary acidification with ammonium chloride (1 g) twice daily is advised, following the guidelines set by the EAU. These measures are aimed at preventing further stone formation and managing the condition effectively.

## 5 Discussion

Urinary stones can be classified based on various criteria, including their size, location within the urinary tract (such as the upper, middle, or lower calyx; renal pelvis; upper, middle, or distal ureter; and urinary bladder), x‐ray characteristics, the etiology of their formation, composition, and the likelihood of recurrence [1]. Calcium stones are significantly more prevalent, accounting for 88% of all types and leading to the development of a condition called “nephrocalcinosis.” [2]. The term “infection stones” refers to calculi that develop after urinary tract infections (UTIs) caused by urease‐producing gram‐negative organisms. These stones are composed of magnesium ammonium phosphate (struvite), carbonate apatite, and monoammonium urate. The formation of these stones is most favorable in alkaline urine conditions [3]. While struvite stones account for only approximately 2%–3% of the stones submitted to the laboratory for analysis, they present more significant clinical issues, including sepsis and potential renal failure, compared to any other type of stone [4].

The treatment options for stones include extracorporeal shock wave lithotripsy (ESWL), ureterorenoscopy (URS), percutaneous nephrolithotomy (PCNL), laparoscopy, and open surgery. Advances in ESWL and endourological surgery (URS and PCNL) have significantly decreased the indications for open or laparoscopic stone surgery [5, 6].

There is a consensus that most complex stones, including partial and complete staghorn stones, should be approached primarily with PCNL. Additionally, a combined approach, known as endoscopic combined intrarenal surgery (ECIRS), which integrates PCNL with retrograde intrarenal surgery (RIRS), may also be an appropriate alternative. However, if percutaneous approaches are not likely to be successful, or if multiple endourological approaches have been performed unsuccessfully; open or laparoscopic surgery may be a valid treatment option [7, 8, 9, 10].

Because of the size and localization of the stone in our patient, the laparoscopic approach has been preferred over all other options. Almost all comparative studies and reported cases in the literature deal with ureteral or pelvic stones. No case of an intraparenchymal stones treated with laparoscopic nephrolithotomy (LNL) has been reported up to date.

Few studies have reported laparoscopic stone removal. A randomized controlled trial conducted by Wang et al. between January 2012 and December 2015 compared mini‐percutaneous nephrolithotomy (MPCNL), URSL, and retroperitoneal laparoscopic ureterolithotomy (RPLU), and evaluated which one is the best choice for large upper impacted ureteral stones. In total, 150 consecutively enrolled patients with a large upper impacted ureteral stone (> 15 mm) were included. Fifteen patients needed auxiliary ESWL after URSL, and three patients after MPCNL, but none after RPLU. The stone clearance rate was 96% (48/50) in the MPCNL group and 72% (33/46) in the URSL group. In the RPLU group, the stones were completely removed and the stone clearance rate was 100% (48/48). MPCNL and RPUL are more suitable for upper ureteral impacted stones of > 15 mm. It concluded that MPCNL and RPUL are more suitable for upper ureteral impacted stones with a diameter of > 15 mm. URSL could be considered if the patient is not suitable for general anesthesia, or the patient requests transurethral ureteroscopic surgery [11].

In a meta‐analysis conducted by Bai et al., five randomized and nine non‐randomized studies were identified for analysis, involving a total of 901 patients to compare PCNL with LPL in terms of efficacy and safety for the management of large renal pelvic stones. There were no significant differences in conversion to open surgery and prolonged urine leakage rates between LPL and PCNL. The findings suggested that LPL is a safe and effective approach for management of patients with large renal stones. However, PCNL is still suitable for most cases and LPL can be used as an alternative management procedure with good selection of cases [12].

A systematic review and meta‐analysis of randomized controlled trials by Torricelli et al. [10] comparing semi‐rigid URS with laparoscopic ureterolithotomy (LUL) for the treatment of the large proximal ureteral stone concluded that LUL for larger proximal ureteral calculi are favorable compared to semi‐rigid URS and should be considered as a first‐line alternative if flexible ureteroscopy is not available.

A few studies with limited numbers of patients have reported using robotic surgery in the treatment of urinary stones.

In a meta‐analysis realized by Müller et al., the benefits of robotic devices in stone surgery in existing endourological, laparoscopic, and open treatment strategies still need elucidation. Although recent data are promising, more prospective randomized controlled studies are necessary to clarify the impact of this technique on patient safety and stone‐free rates [13].

The EAU recommend open surgery as the last treatment option, after all other possibilities have been explored. Studies on laparoscopy should be interpreted with caution due to their poor design and low quality of evidence. It recommends however to offer laparoscopic or open surgical stone removal in rare cases in which shock wave lithotripsy, retrograde or antegrade ureteroscopy and percutaneous nephrolithotomy fail, or are unlikely to be successful [1].

Regarding infection stones, the European Association of Urology (EAU) recommends both general and specific preventive measures. General preventive measures involve promoting adequate fluid intake and maintaining a healthy diet. Specific measures, on the other hand, include complete surgical removal of the stones, short‐ or long‐term antibiotic treatment, urinary acidification through the use of methionine or ammonium chloride, and providing advice to restrict intake of urease. All of these approaches are aimed at reducing the risk of stone formation and recurrence in patients with infection stones [1].

## Author Contributions

**Ayoub Hidayat‐Allah:** conceptualization, data curation, methodology, resources, writing – original draft, writing – review and editing. **Frank Friedersdorff:** supervision, validation.

## Ethics Statement

IRB approval was obtained before proceeding to study.

## Consent

Written informed consent was obtained from the patient to publish this report in compliance with the journal's patient consent policy, ensuring that the patient has granted permission for their clinical information and photographic material to be included in the publication.

## Conflicts of Interest

The authors declare no conflicts of interest.

## Data Availability

The datasets analyzed during the current study are not publicly available due to privacy concerns and institutional restrictions, but are available from the corresponding author upon reasonable request.
